# Superior oblique myositis mimics a subperiosteal abscess in a patient with sinusitis

**DOI:** 10.5935/0004-2749.20210078

**Published:** 2021

**Authors:** Muhannad I. AlKhalifah, Mohammad O. Aloulah, Yasser H. Al-Faky

**Affiliations:** 1 Department of Ophthalmology, College of Medicine, King Saud Medical City, King Saud University, Riyadh, Saudi Arabia.; 2 Department of Otolaryngology, College of Medicine, King Saud Medical City, King Saud University, Riyadh, Saudi Arabia

**Keywords:** Myositis/diagnostic imaging, Sinusitis, Orbital disease, Oculomotor muscle, Human, Case report, Miosite/diagnóstico por imagem, Sinusite, Doenças orbitárias, Músculos oculomotores, Humanos, Relatos de casos

## Abstract

Isolated superior oblique myositis is a rare variant of idiopathic orbital myositis. We are reporting for the first time the case of a 19-year-old woman who had isolated superior oblique myositis with sinusitis that mimics a subperiosteal abscess. Despite the typical history of upper respiratory tract infection and laboratory test results and initial radiological findings suggestive of orbital cellulitis secondary to sinusitis, the initial response to systemic steroid with subsequent imaging changes and the relapse after cessation of steroid therapy helped us reach the diagnosis.

## Abstract (pt)

A miosite oblíqua superior isolada é uma variante muito rara da miosite orbital idiopática. Trata-se do primeiro relato de uma mulher de 19 anos como um caso de miosite oblíqua superior isolada com sinusite que mimetiza abscesso subperiosteal. Apesar da história típica de infecção do trato respiratório superior, exames laboratoriais e achados radiológicos iniciais sugestivos de celulite orbital secundária à sinusite, a resposta inicial ao esteróide sistêmico com subsequentes alterações de imagem e recaída, após a cessação do esteróide, nos ajudou a alcançar o diagnóstico.

## INTRODUCTION

Idiopathic orbital myositis is the second most common extraocular muscle disease after thyroid associated myopathy^(1)^. The etiological factors of idiopathic orbital myositis are not fully understood, but associated conditions were reported in the literature, including paranasal sinus diseases^(2)^. Isolated superior oblique involvement as a variant of idiopathic orbital inflammatory syndrome is rare^(1)^. To the best of our knowledge, this is the first report of isolated superior oblique myositis with sinusitis that mimics a subperiosteal abscess.

## CASE REPORT

A 19-year-old woman was referred to our tertiary hospital as a case of right orbital cellulitis that showed no improvement with systemic antibiotics (vancomycin, ceftriaxone, and metronidazole) for 4 days. The patient complained of right acute-onset progressive deep orbital pain associated with periorbital swelling. She reported a history of upper respiratory tract infection with nasal discharge and frontal pain over the previous week. She had never encountered similar symptoms, and her past ocular and medical history were unremarkable.

**Figure 1:**
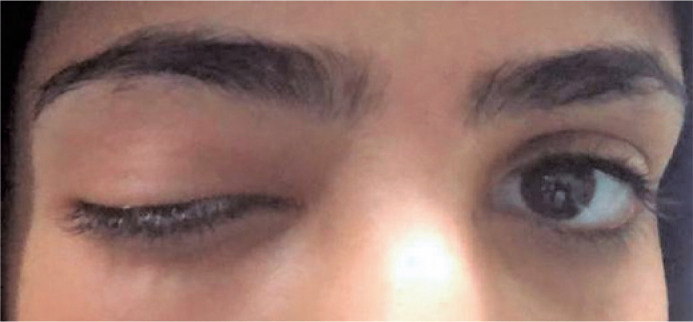
External photo of the patient.

On examination, the patient was afebrile and had right upper eyelid significant ptosis (Figure 1) with inferotemporal globe displacement. Extraocular muscle motility examination revealed limitation of depression and elevation on adduction of the right eye, at which the patient reported both horizontal and vertical diplopia. The visual acuity was 20/20, with normal color vision and pupillary reaction in both eyes. The left orbital examination result was unremarkable. A complete blood count showed a normal white blood cell count with increased neutrophil percentage and high C-reactive protein level, which were suggestive of bacterial infection.

**Figure 2:**
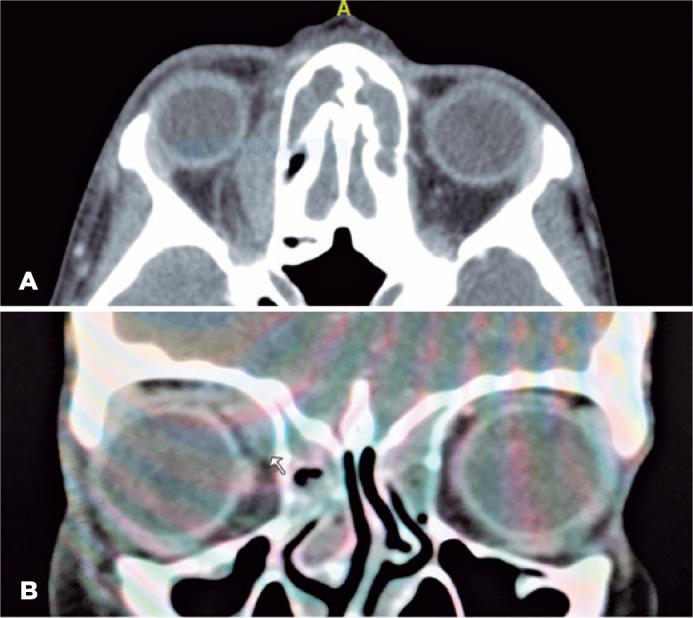
CT scan of the orbits upon admission. A) Axial superior orbital

A computed tomography scan with contrast of the orbits and paranasal sinuses demonstrated pan-sinusitis and was suggestive of superomedial subperiosteal abscess in the right orbit (Figure 2). The patient was admitted for medical management and possible combined endoscopic sinus surgery and drainage of the subperiosteal abscess. Upon admission, she received intravenous administration of ceftriaxone and clindamycin in addition to dexamethasone 8 mg at admission and two more doses every 12 hours.

**Figure 3:**
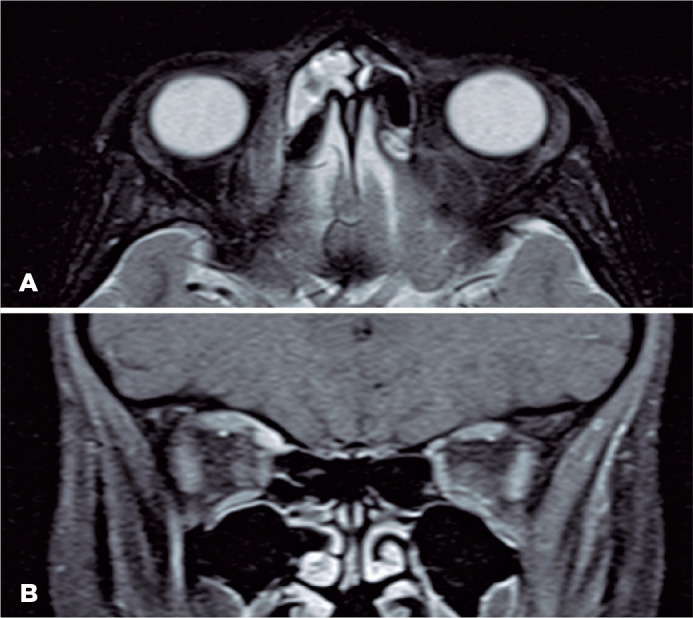
MRI of the orbits. A) Axial T2 without contrast and fusiform encut shows enlarged superior oblique myositis mimicking a subperiosteal largement involving the whole right superior oblique muscle. B) Coronal abscess, B) The coronal orbital cut shows the superomedial soft tissue T1 image with contrast showing the enlargement of the right superior density with ethmoidal sinusitis. oblique muscle with enhancements of the gadolinium contrast.

After 24 hours, the patient’s condition dramatically improved on medical therapy, and surgical intervention was deferred while keeping the patient on antibiotic therapy. Two days later, after cessation of the corticoste roid therapy, the symptoms recurred with gradual worsening of the pain, diplopia, and swelling. At this stage, magnetic resonance imaging (MRI) was ordered and revealed improvement of the sinusitis along with right superior oblique muscle and tendon enlargement and enhancement. A clear demarcation was observed between the muscle and the non-elevated medial orbital wall with no fluid collection (Figure 3). Hence, isolated right superior oblique myositis was diagnosed. At this stage, measurement of IgG4 level was ordered, and the result was unremarkable.

Oral prednisolone therapy was started at 1 mg/kg/day and favorable response was observed in <24 hours. The patient remained admitted for 3 more days for observation. At the time of discharge, the patient was symptom-free with complete resolution of the swelling and pain; however, residual limitation of elevation on adduction (inflammatory Brown syndrome) was still noticed. At the 4-week follow-up visit, the patient fully recovered without pain, swelling, or ocular motility limitations, and systemic steroid was gradually withdrawn over 3 months.

## DISCUSSION

Gleason first described idiopathic orbital myositis in 1903 as a benign inflammation of unknown etiology^(3)^. Though uncommon, upper respiratory tract infection and sinusitis were reported as potential triggers of idiopathic orbital myositis^(2,4)^. The clinical presentation of the disease is variable, and the cardinal feature is deep orbital pain aggravated by eye movement. Other findings include mild periorbital and lid edema, ptosis, diplopia with limited ocular motility along the vector of the affected muscle, conjunctival chemosis, and minimal proptosis^(1)^. In our case, the patient had all the previously mentioned features.

The largest reported idiopathic orbital myositis series by Siatkowski et al. indicated that 68% of patients have only one muscle affected^(5)^. The frequently affected muscles are the lateral rectus (33%), medial rectus (29%), and superior rectus (23%), while the superior oblique muscle was the least frequently affected (2%). Yan and Wu reported that only 1.16% of the patients in their series had superior oblique myositis^(6)^. Other studies reported superior oblique myositis as a manifestation of Sjogren syndrome^(7,8)^. To our knowledge, 2 cases of orbital myositis associated with sinusitis were published in the English literature, but none of them occurred in the superior oblique muscle. The first case reported by Dylewski et al. involved the medial rectus muscle, while Neumann et al. reported a case involving the lateral rectus muscle^(2,4)^.

In our report, the clinical presentation with the initial CT findings masqueraded the superior oblique myositis as a subperosteal abscess; hence, the case was labeled as such. The close approximity of the superior oblique muscle to the medial orbital wall with the intense inflammation made it difficult to identify distinct borders between the structures and misled the diagnosis to subperiosteal abscess. However, the dramatic response to the initial dexamethasone dose and the relapse after its effect has waned influenced our management plan, and further imaging confirmed the diagnosis of myositis. The use of systemic steroid in orbital cellulitis is controversial as the final outcomes are almost the same. Some authors recommend its use from the start to shorten the hospital stay and hasten the recovery, while others find it unsafe to suppress the patient’s immunity under an infectious etiology^(9)^. In this case, it was advantageous to use a short-term systemic steroid therapy that helped us to diagnose superior oblique myositis.

The treatment of choice in orbital myositis is corticosteroids, being the most commonly used initial therapy, with oral prednisolone 1 mg/kg/day for 2 weeks followed by slow tapering ^(5)^. It is not uncommon for steroid monotherapy to fail to control the disease and to be associated with more relapses. Therefore, for the recurrent cases, immunosuppresants and biologics have proven effective as steroid-sparing agents^(1,5)^.

Isolated superior oblique myositis is a rare form of orbital myositis. The clinical presentation and radiological findings may mimic, though rarely, other orbital pathologies such as subperiosteal abscess. Hence, practicing ophthalmologists should consider the diagnosis of orbital myositis in cases of atypical presentation of orbital celluitis or those are unresponsive to antimicrobial therapy.
